# Use of brimonidine tartrate to resolve telangiectatic matting: case report

**DOI:** 10.1590/1677-5449.190159

**Published:** 2020-09-14

**Authors:** Brenno Augusto Seabra de Mello, Yasmin de Rezende Beiriz, Américo Carnelli Bonatto, Gustavo Sasso Benso Maciel, Laila Reggiani de Almeida, José Marcelo Corassa

**Affiliations:** 1 Venno Clinic - Excelência Vascular, Departamento de Cirurgia Vascular, Vitória, ES, Brasil.; 2 Escola Superior de Ciências da Santa Casa de Misericórdia de Vitória – EMESCAM, Curso de Graduação em Medicina, Vitória, ES, Brasil.; 3 Faculdade Brasileira MULTIVIX, Curso de Graduação em Medicina, Vitória, ES, Brasil.

**Keywords:** sclerotherapy, telangiectasis, varicose veins. case report

## Abstract

Sclerotherapy is currently the treatment of choice for telangiectasias and reticular veins, with grade 1A recommendation in the European Guideline for sclerotherapy. The most common side effects of this procedure are hyperpigmentation and telangiectatic matting, the second of which provokes great concern because of the esthetic damage and the difficulty of treatment. Matting refers to vessels with a diameter of less than 0.2 mm, which may emerge irregularly or in well-defined areas, especially on the lower limbs. This report presents a case of matting treated with topical Brimonidine Tartrate.

## INTRODUCTION

Venous diseases are classified on the basis of clinical data (C), etiology (E), anatomic distribution (A) and pathophysiology (P), using the CEAP classification.^1^ Telangiectasias and reticular veins are grouped in class 1 (C1) of the CEAP clinical classification,^2^ and the treatment of choice for telangiectasias is sclerotherapy.^3^

Currently, polidocanol and hypertonic glucose are the most widely used sclerosants.^4^ However, each treatment is associated with a series of complications, and the most common side effects of sclerotherapy are hyperpigmentation and matting.^4^

Telangiectatic matting is one of the complications that causes greatest concern because of the esthetic damage and the difficulty of treatment. Matting is formed by vessels with diameters of less than 0.2 mm that can appear irregularly or in well-defined areas, primarily on the lower limbs.^5^ Angiogenesis and vasodilation are factors related to matting, although no definitive cause has been fully established.^6^ However, some hypotheses have been raised, including one based on estrogen and another founded on local inflammatory reactions.

It is known that endothelial cells have estrogen receptors, which suggests that endogenous and exogenous estrogen plays a role in angiogenesis, encouraging the emergence of matting. Additionally, inflammatory factors such as fibronectin can attack the basement membrane of the endothelium, which can induce angiogenesis.^7^

This report presents a case of matting that was resolved macroscopically using topical brimonidine tartrate.

## CASE DESCRIPTION

A 19-year-old female patient, with Fitzpatrick II skin color, who was a non-smoker, sedentary, free from comorbidities or drug allergies, and was taking oral contraception regularly, presented with an esthetic complaint of combination telangiectasias on the lateral surface of the left thigh. She was told that she needed to use skin moisturizer and take pycnogenol 200 mg/day orally for at least 15 days before undergoing a procedure. Detailed photographic records were taken and the patient was given a consent form with detailed information about the treatment and its possible complications. When she returned 15 days later, the patient underwent conventional sclerotherapy of the combination telangiectasias on the lateral surface of her left thigh, using a solution containing glucose 65% and polidocanol 0.5% (to a total volume of 1.5 mL).

**Figure 1:**
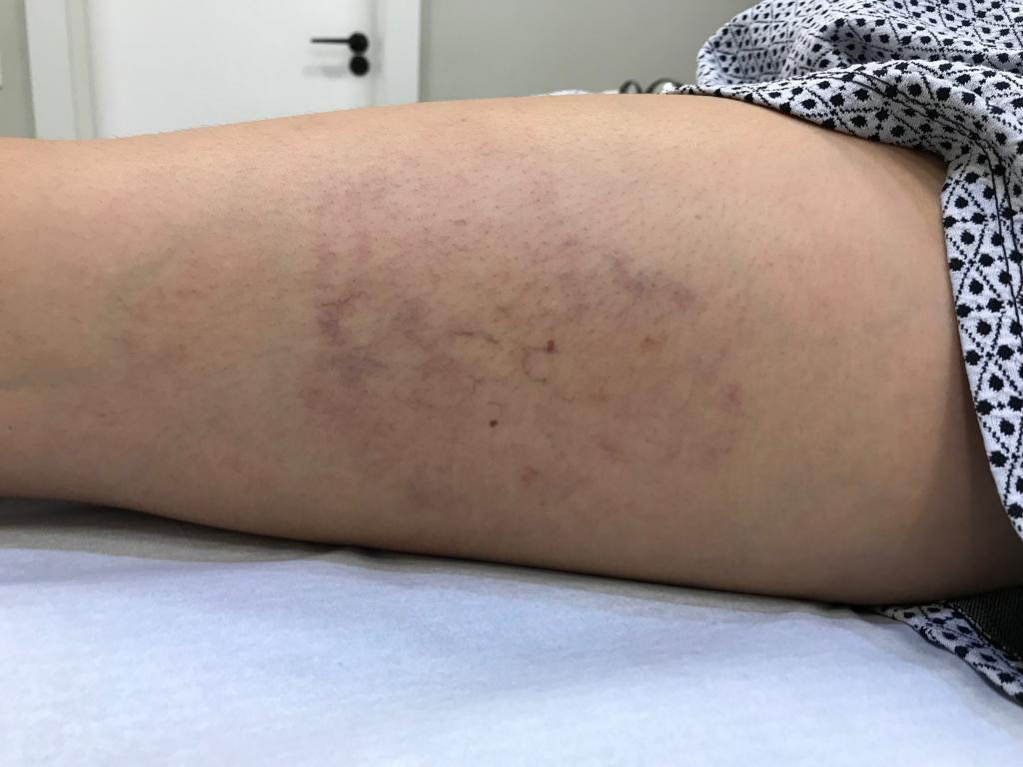
Two days after sclerotherapy, with telangiectatic matting .

She developed a large expanse of telangiectatic matting (approximately 10 x 15 cm) in the area surrounding the vessels that had been treated, about 2 days after the procedure (Figure 1). An augmented reality venous Doppler ultrasound examination of the region did not show varicose veins or feeder veins that could be linked with the complication.

**Figure 2:**
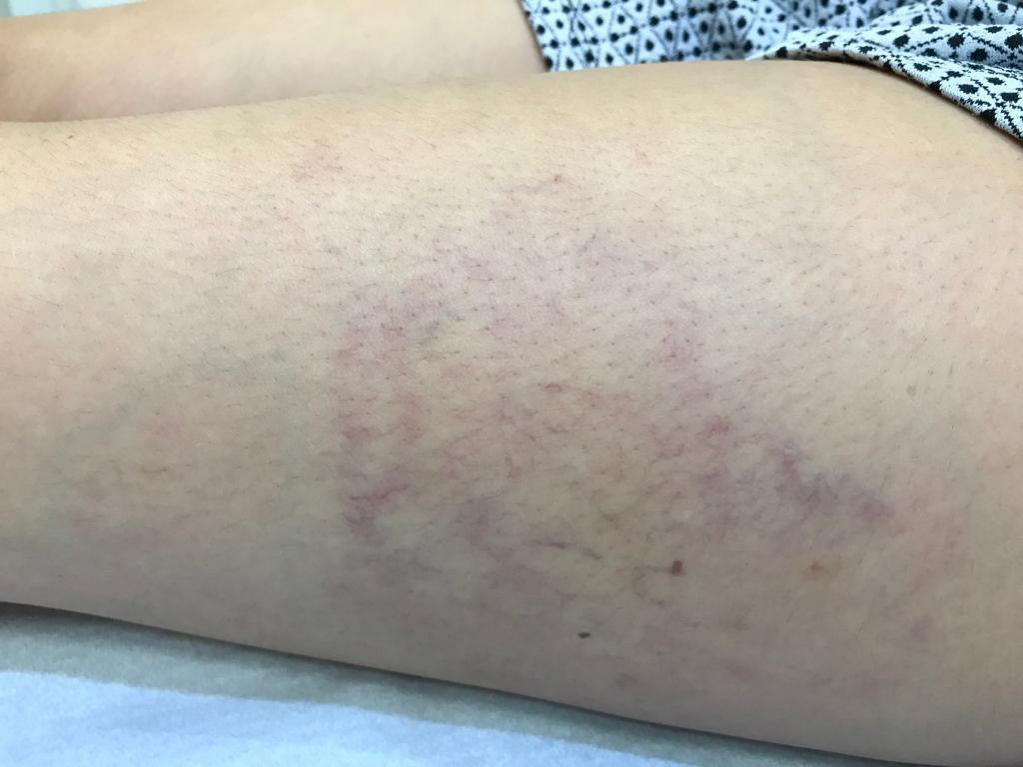
Unsatisfactory results after attempt to treat with intense pulsed light.

Initial management consisted of reassuring the patient and instructing her not to expose herself to sunlight and prescribing oral and topical pycnogenol. Additionally, three sessions of intense pulsed light were administered at 540 nm/17 J/15 ms (similar parameters to those used to treat rosacea), with 21-day intervals between sessions. In the opinions of the same evaluator and of the patient, there was no more than mild attenuation of the condition (Figure 2).

**Figure 3:**
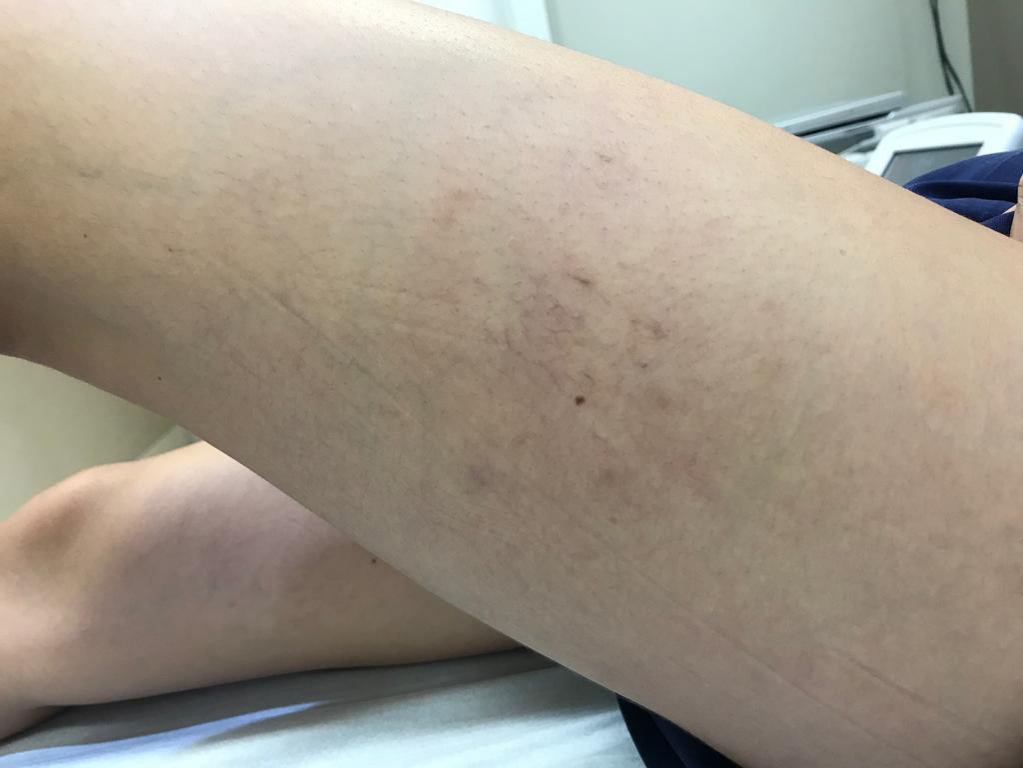
Appearance after 1 week using brimonidine tartrate.

**Figure 4:**
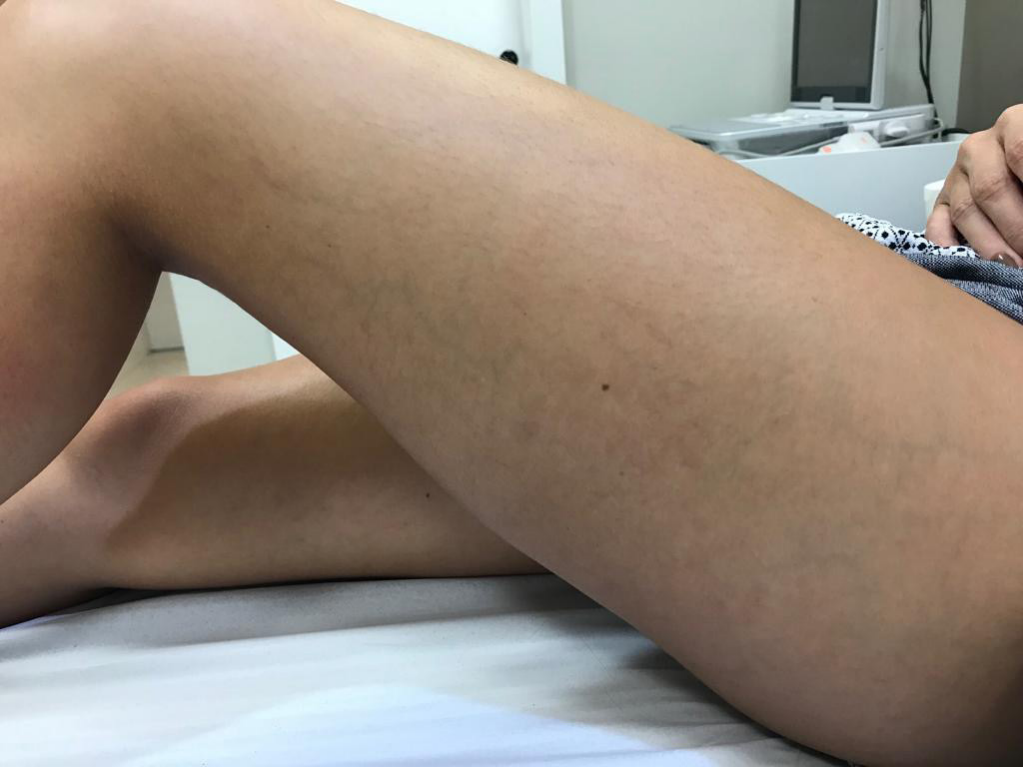
Result after 2 weeks using brimonidine tartrate.

**Figure 5:**
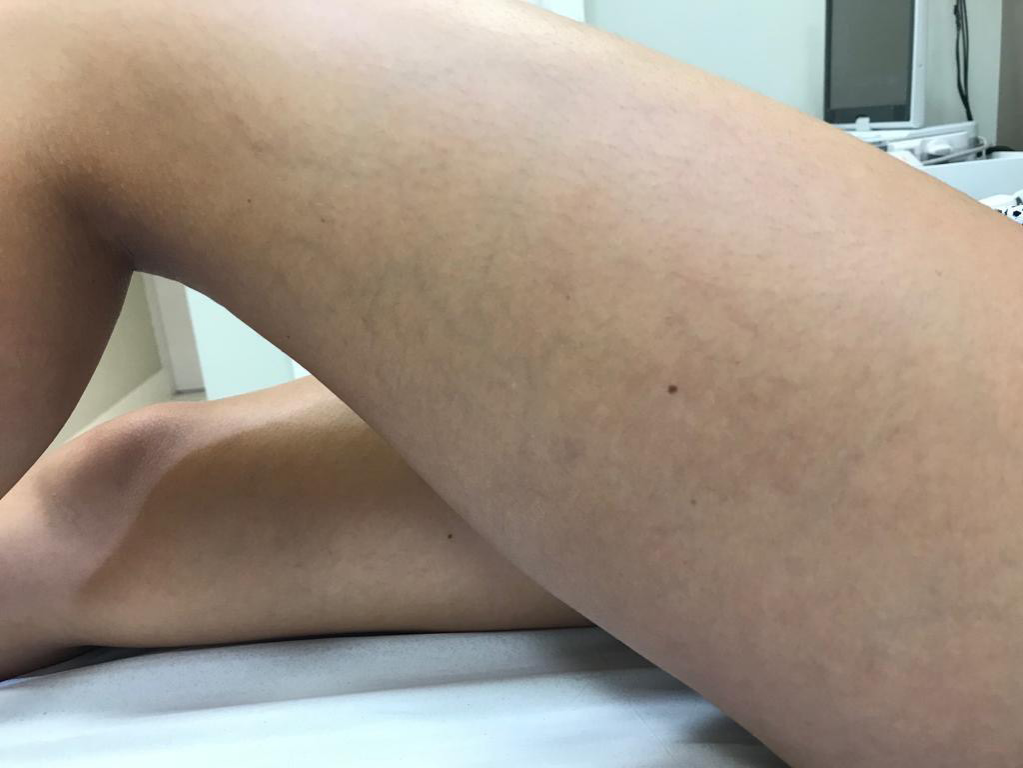
Follow-up, 6 months after appearance of telangiectatic matting.

In view of this, the patient was instructed to administer topical brimonidine tartrate 0.5% daily. After 7 days of regular use of this medication, considerable improvement was observed (Figure 3). A joint decision was taken to continue applying the medication for a further 7 days, with very satisfactory results (Figure 4). After 14 days’ use, we withdrew the medication and continued the follow the patient up clinically. She is satisfied and both the telangiectasias and the telangiectatic matting have completely disappeared. The initiative to treat with brimonidine tartrate was based on use of Mirvaso® to treat rosacea, which has a pathophysiology that has certain similarities with matting. Because it is difficult to source, the medication was ordered from a compounding pharmacy. The patient has been in clinical follow-up for 6 months (Figure 5) and no rebound effect has been observed to date.

## DISCUSSION

The etiology of telangiectatic matting is unknown, but it is known that it is more common in women and that risk factors include family history of telangiectasia, excess of exogenous female hormones, and obesity.^8^ Post sclerotherapy matting occurs after approximately 15 to 20% of treatments and consists of appearance of small red telangiectasias in the vicinity of the treated vein.^4^ It is characterized by emergence of irregular pigmentation with onset from 4 to 6 weeks after treatment. Technical measures employed to avoid this complication include use of minimal sclerosant concentrations, low volumes, and low pressure during sclerotherapy.^4^ Additionally, classifying the patient’s Fitzpatrick skin phototype and moisturizing with pycnogenol in preparation for the procedure are measures that can induce better outcomes.

Matting may be transitory (with spontaneous resolution from 3 to 12 months after treatment), but it can also be permanent.^9^ Initial treatment is based on locating untreated proximal reflux in saphenous veins, perforators, tributaries, or reticular veins.^10^ Investigating associated comorbidities is very important for defining the etiology underlying the reaction, since an increased tendency to development of matting is observed in allergic patients and those with bleeding disorders, a diagnosis of bronchial asthma, or ongoing hormone therapy.^5^

Careful choice of the sclerosing agent, care with puncture technique and selection of vessels for treatment, and also sessions employing smaller volumes and lower pressures may help avoid negative results. In the current state of the art of phlebology, no effective topical treatment for the vasomotor changes involved in matting is known. The clinical relevance of this case report primarily lies in the description of an alternative topical treatment for matting. Options using lasers, such as intense pulsed light or Nd YAG 1064 supplement the therapeutic arsenal and have their place for patients with puncture phobia or as adjuvants.^11^

It is known that the potency of the solution used for sclerotherapy can be a causative factor of matting. Use of hypertonic glucose alone may possibly be a factor in avoiding the complication described here. Regardless, this case report aims to analyze the possibility of treatment of a complication, which, in this case, is telangiectatic matting.

In this situation, brimonidine tartrate, which is usually used for rosacea, emerged as an alternative treatment for telangiectatic matting. This medication is a selective alpha-2 adrenergic agonist.^12^ It has proved safe when used topically, with acceptable tolerability, and has not been linked with side effects of significant severity.^12^

Erin Lowe^13^ described appearance of paradoxical erythema reaction with prolonged use in rosacea cases, which was resolved by withdrawal of the treatment for 48 hours, with full resolution of the rash. Detailed explanations of the risks of the procedure, esthetic refinements, clarity, knowledge of treatments, and a good doctor-patient relationship are indispensable for patient compliance with treatment that is difficult and can be expensive.

In the matting case described here, use of brimonidine tartrate resolved the patient’s complaint without provoking side effects. A topical treatment option for secondary telangiectasia could offer new prospects for phlebology. While this medication may constitute a new option for treatment of telangiectatic matting, prospective clinical studies with sample size calculations should be conducted to yield additional scientific evidence on this application and to investigate the safety of the treatment for this specific condition.
