# Supplementary material for: Use of brimonidine tartrate to resolve telangiectatic matting: case report
Source: J Vasc Bras. 2020 Sep 14;19:e20190159. doi: 10.1590/1677-5449.190159 (PMC8276647; doi:10.1590/1677-5449.190159)
Supplement: Translation

# Translation: Uso de tartarato de brimonidina para resolução de *matting* telangiectásico: relato de caso

**Authors:** Brenno Augusto Seabra de Mello, Yasmin de Rezende Beiriz, Américo Carnelli Bonatto, Gustavo Sasso Benso Maciel, Laila Reggiani de Almeida, José Marcelo Corassa

**Affiliations:** 1 Venno Clinic - Excelência Vascular, Departamento de Cirurgia Vascular, Vitória, ES, Brasil.; 2 Escola Superior de Ciências da Santa Casa de Misericórdia de Vitória – EMESCAM, Curso de Graduação em Medicina, Vitória, ES, Brasil.; 3 Faculdade Brasileira MULTIVIX, Curso de Graduação em Medicina, Vitória, ES, Brasil.

**Keywords:** escleroterapia, telangiectasia, varizes, relato de caso

## Abstract

A escleroterapia é, atualmente, o tratamento de escolha para telangiectasias e veias reticulares, apresentando nível de recomendação 1ª pela diretriz europeia para escleroterapia. Os efeitos colaterais mais comuns desse procedimento são a hiperpigmentação e o *matting* telangiectásico, sendo este último um dos mais temidos em virtude do dano estético e da dificuldade de tratamento. O *matting* se refere a vasos com diâmetro inferior a 0,2 mm que podem surgir esporadicamente ou em áreas bem definidas, principalmente nos membros inferiores. Este relato apresenta um caso de *matting* tratado com o uso de tartarato de brimonidina tópico.

## INTRODUÇÃO

A classificação das doenças venosas é baseada em dados clínicos ©, etiologia (E), distribuição anatômica (A) e fisiopatologia (P), denominada classificação CEAP^1^. As telangiectasias e as veias reticulares compreendem a classe 1 (C1) da classificação clínica de CEAP^2^, e o tratamento de escolha para as telangiectasias é a escleroterapia^3^.

Atualmente, os esclerosantes mais utilizados são polidocanol e glicose hipertônica^4^. Contudo, cada tratamento apresenta uma série de complicações, sendo os efeitos colaterais mais comuns da escleroterapia a hiperpigmentação e o *matting* ^4^.

Nesse contexto, o *matting* telangiectásico, também conhecido como nuvem telangiectásica, é um dos mais temidos pelo dano estético e pela dificuldade de tratamento. O *matting* se refere a vasos com diâmetro inferior a 0,2 mm que podem surgir esporadicamente ou em áreas bem definidas, principalmente nos membros inferiores^5^. A angiogênese e a vasodilatação são fatores associados ao *matting,* ainda que não se tenha uma causa plenamente estabelecida^6^. Assim, algumas hipóteses foram criadas, como a do estrogênio e a das reações inflamatórias locais.

Sabe-se que as células endoteliais apresentam receptores de estrogênio, o que sugere que o estrogênio endógeno e exógeno apresenta um papel na angiogênese, favorecendo o aparecimento de *matting*. Além disso, fatores inflamatórios como a fibronectina podem atacar a membrana basal do endotélio, podendo levar à angiogênese^7^.

O relato a seguir apresenta um caso de *matting* macroscopicamente resolvido com o uso de tartarato de brimonidina tópico.

## DESCRIÇÃO DO CASO

Uma paciente feminina de 19 anos, Fitzpatrick II, não tabagista, sedentária, sem comorbidades ou alergia medicamentosa e em uso regular de anticoncepcional oral​ apresentava queixa estética motivada por telangiectasias combinadas na face lateral da coxa esquerda. Foi orientada quanto à necessidade de hidratação cutânea e uso de picnogenol 200 mg/dia por via oral por pelo menos 15 dias antes de passar por procedimento. Foi realizada fotodocumentação detalhada, e a paciente recebeu o termo de consentimento com informações detalhadas do tratamento e possíveis complicações. Ao retornar após 15 dias, a paciente foi submetida a escleroterapia convencional em telangiectasias combinadas na face lateral da coxa esquerda, com solução de glicose 65% e polidocanol 0,5% (volume total de 1,5 mL).

Na área que margeava os vasos de interesse, desenvolveu nuvem telangiectásica de grandes dimensões (10 x 15 cm aproximadamente) cerca de 2 dias após o procedimento (Figura 1). No exame de realidade aumentada e de eco-Doppler venoso da região, não foram visualizadas varizes ou veias nutridoras que poderiam ter associação com a complicação em questão.

**Figura 1:** Dois dias após escleroterapia, com surgimento de *matting* telangiectásico.

A conduta inicial contemplava, além de tranquilizar a paciente, manter a não exposição solar e o uso de picnogenol oral e tópico. Foram realizadas, ainda, três sessões de luz intensa pulsada 540 nm/17 J/15 ms (parâmetros similares ao tratamento de rosácea), com intervalo de 21 dias entre as sessões. Aos olhos do mesmo avaliador e da paciente, houve apenas leve atenuação do aspecto (Figura 2).

**Figura 2:** Resultado insatisfatório após tentativa de tratamento com luz intensa pulsada.

Portanto, foi proposto o uso de tartarato de brimonidina 0,5% por via tópica com uma aplicação diária. Após 7 dias do uso regular da medicação, houve melhora considerável da lesão (Figura 3). Optamos em conjunto por continuar por mais 7 dias, com resultado muito satisfatório (Figura 4). Após 14 dias, suspendemos o uso da medicação e seguimos acompanhando apenas clinicamente a paciente, que se encontra satisfeita, com completo desaparecimento das telangiectasias e do *matting* telangiectásico. A iniciativa de tratamento com tartarato de brimonidina foi baseada no uso do Mirvaso®, medicamento utilizado para rosácea cuja fisiopatologia possui algumas similaridades com o *matting.* Em virtude da dificuldade de encontrar esse medicamento, optou-se pela manipulação da medicação. A paciente encontra-se em seguimento clínico há 6 meses (Figura 5), e não foi observado efeito rebote até o momento.

**Figura 3:** Aspecto da lesão após 1 semana do uso de tartarato de brimonidina.

**Figura 4:** Resultado após 2 semanas do uso do tartarato de brimonidina.

**Figura 5:** Acompanhamento em 6 meses após aparecimento do *matting* telangiectásico.

## DISCUSSÃO

A etiologia do *matting* telangiectásico é desconhecida, mas sabe-se que ocorre mais em mulheres e apresenta, como fatores de risco, história familiar de telangiectasia, excesso de hormônios femininos exógenos e obesidade^8^. O *matting* pós-escleroterapia ocorre em aproximadamente 15 a 20% dos tratamentos e consiste no aparecimento de pequenas telangiectasias vermelhas na área da veia tratada^4^. Ele se caracteriza pelo surgimento de uma pigmentação irregular de início em 4 a 6 semanas após o tratamento. Utilizar concentração mínima de esclerosante, pequenos volumes e baixa pressão durante a escleroterapia são medidas técnicas para evitar essa complicação^4^. Outrossim, classificar o fototipo cutâneo segundo Fitzpatrick e fazer hidratação com picnogenol pré-procedimento são cuidados que podem promover melhores desfechos.

O *matting* pode ser transitório (com resolução espontânea de 3 a 12 meses após o tratamento) ou permanente^9^. O tratamento inicial é baseado em localizar o refluxo proximal não tratado de veias safenas, perfurantes, tributárias ou reticulares^10^. Investigar comorbidades associadas é de grande importância para definição da etiologia da reação, uma vez que se observou maior tendência ao desenvolvimento de *matting* nos pacientes alérgicos, com distúrbio de sangramento, diagnóstico de asma brônquica ou terapia hormonal em curso^5^.

Um olhar atento sobre a escolha do esclerosante, cuidado na técnica de punção e seleção dos vasos de interesse, bem como sessões com menor volume e pressão, podem driblar o resultado negativo. No cenário atual da flebologia, não se conhece um tratamento por via tópica eficaz para as alterações vasomotoras do *matting*. A relevância clínica deste relato de caso está principalmente relacionada a um tratamento tópico alternativo para o tratamento do *matting.* As opções de *laser* como luz intensa pulsada ou Nd YAG 1064 vêm para complementar o arsenal terapêutico e têm seu espaço nos casos de pacientes com fobia de punção ou em adjuvância^11^.

Sabe-se que a potência da solução utilizada para escleroterapia pode ser um fator causador do *matting.* O uso de glicose hipertônica isolada poderia, talvez, ser um fator para evitar a complicação apresentada. Todavia, este relato de caso objetiva analisar uma possibilidade de tratamento da complicação que, no caso, é o *matting* telangiectásico.

Diante desse cenário, o tartarato de brimonidina, usualmente utilizado para rosácea, surge como um tratamento alternativo para o *matting* telangiectásico. Esse medicamento é um agonista alfa-2 adrenérgico seletivo^12^. Seu uso tópico se mostrou seguro e com adequada tolerabilidade, não apresentando efeitos colaterais de grande magnitude^12^.

Erin Lowe^13^ descreveu o aparecimento de eritema paradoxal reacional com uso prolongado nos casos de rosácea, o que foi resolvido com descontinuação do tratamento por 48 horas, com completa resolução do quadro de *rash*. Explicações detalhadas sobre os riscos do procedimento, refinamento estético, clareza, conhecimento sobre os tratamentos e boa relação médico-paciente são indispensáveis para a adesão do paciente a um tratamento difícil e, às vezes, oneroso.

O uso de tartarato de brimonidina no caso de *matting* descrito trouxe resolução para a queixa da paciente, sem apresentar efeitos colaterais. Uma opção de tratamento tópico para telangiectasia secundária pode acrescentar novas perspectivas para a flebologia. Embora essa medicação possa ser uma nova opção para o tratamento do *matting* telangiectásico, estudos clínicos prospectivos com amostra calculada devem ser realizados para que se possa obter maior evidência científica dessa indicação de uso, além de averiguar a segurança do tratamento para essa condição específica.
